# How to Measure Retirement Age? A Comparison of Survey and Register Data

**DOI:** 10.1007/s12062-019-09254-6

**Published:** 2019-10-24

**Authors:** Harpa S. Eyjólfsdóttir, Isabel Baumann, Neda Agahi, Carin Lennartsson

**Affiliations:** 1 Aging Research Center, Karolinska Institutet and Stockholm University, Tomtebodavägen 18 A, 171 65 Solna, Sweden; 2 Center for Health Sciences, Zurich University of Applied Sciences, Technikumstrasse 81, 8400 Winterthur, Switzerland; 3 National Center of Competence in Research “Overcoming Vulnerability: Life Course Perspectives”, Geneva, Switzerland

**Keywords:** Retirement transition, Retirement age, Old-age pensions, Measurement, Survey data, Register data

## Abstract

Due to an increasing heterogeneity in retirement transitions, the measurement of retirement age constitutes a major challenge for researchers and policymakers. In order to better understand the concept of retirement age, we compare a series of measures for retirement age assessed on the basis of survey and register data. We use data from Sweden, where flexible retirement schemes are implemented and register data are available. We link survey data from the Swedish Level of Living Survey with register data from the Swedish Longitudinal Integration Database for Health Insurance and Labour Market Studies. We create four measures of retirement age based on these datasets, applying approaches that have been used in previous literature. We analyse the means and distributions of these measures and evaluate the correlations between them. Finally, we regress common predictors of retirement age such as gender or education on the four measures of retirement age to examine potential differences in size, direction and statistical significance of the associations. We find that the survey measure of retirement age resembles the following two ways of defining retirement age in the register data: first, the age at which people receive more than half their income from old-age or disability pension and, second, the age at which they were not gainfully employed for at least 2 years. This insight gives us a better understanding of when in the retirement transition process, individuals identify with retirement. Moreover, it provides decision support for researchers working with register data to determine which measure to use.

## Introduction

Retirement is one of the most important life course transitions in later life and has gained increasing attention in recent years from both scientists and policy makers. Although retirement is a widely used concept, there does not exist a consensus on how to define and measure it. The countless definitions render the comparison of patterns of retirement over time and between countries challenging. Moreover, retirement in the form of a one-time and complete withdrawal from the labour market is becoming less frequent (Cahill et al. 2015). Instead, the transition from employment to retirement is often gradual or involves alternating decreases and increases of working hours—so-called bridge-employment—sometimes involving changes to new jobs (Beehr and Bennett 2015; Vickerstaff 2010; Wang 2007).

In the literature, the timing of retirement has been defined in various ways—depending amongst other factors on the discipline in which a study was integrated. Broadly speaking, some definitions are based on objective measures such as reducing working hours or receiving a pension from employers or social security, whereas others are based on more subjective measures such as workers’ self-report (J. E. Kim and Moen 2002; Kohli and Rein 1991; Shultz and Wang 2011). In turn, the workers’ self-reported assessments may vary depending on the wording of the survey questions.

In this study, we compare four definitions of retirement age based on survey and register data used in the literature. We draw on a unique dataset combining survey data from the Swedish Level-of-Living Survey (LNU) with register data from the Longitudinal Integration Database for Health Insurance and Labour Market Studies (LISA), linked together on the individual level. Using self-reported retirement age from survey data, and three different definitions of retirement age in register data for the same individuals, we can get a sense of how these four definitions compare to each other and whether they lead to different results when doing research on retirement. More precisely, we address whether (1) average retirement age significantly differs between the four definitions of retirement age and whether (2) common determinants of retirement age are different depending on the definition of retirement age used.

## Literature Review

### The Challenge of Measuring Retirement Age

When covering the topic of retirement in surveys, the assessment of workers’ self-reported labour market participation may be ambiguous (Elder and Pavalko 1993). For instance, respondents might be asked to report year and month of retirement—an event that might have occurred many years before. This process might be susceptible to recall bias. Generally, the more distant in the past an event has taken place, the less accurately it will be recalled (Clarke et al. 2008). A second source of bias, related to social desirability, may be that early retirement is associated with adverse health status (Bender 2012). As a consequence, people who retire early because of poor health may provide an older-than-accurate retirement age to hide this information. Respondents might also be asked to report if they are retired or not retired, but often questionnaires are not detailed enough to capture phenomena such as part-time retirement, bridge employment or reemployment after retirement. Instead, surveys often handle retirement as a complete and final transition from full participation in the labour force to complete cessation of participation (Drobnic 2002). In addition, survey respondents may have a different definition of their retirement age than the researchers, or the definition may even differ between respondents with similar transition to retirement (Cahill et al. 2015). An advantage of using self-reported retirement is that the respondents’ subjectively perceived status is assessed, which might be a better assessment of one’s own perception and identity of retirement status than the objective drop in income or number of hours worked (Denton and Spencer 2009; Lindwall et al. 2017).

Register data are also used to identify retirement age. This has some advantages over using survey data. First, income register data often provides information on the whole population, eliminating selection and item-non response bias and making it possible to study subgroups because of the large sample size (Thygesen and Ersbøll 2014). Second, using register data to identify retirement age allows researchers to be confident that the measurement is created the same way for all study subjects. A downside of register data is that it does not provide subjective measures. An additional limitation is that income register data is usually based on annual reports, which implies that the exact date or month of retirement cannot be assessed.

For several decades, researchers from different disciplines have proposed various definitions and measurements of retirement age, but only three studies have discussed these definitions in detail (Denton and Spencer 2009; Ekerdt and Deviney 1990; Lazear 1986). The most recent of these, Denton and Spencer (2009), analysed Canada-based studies between 1982 and 2007, adding some studies from the USA and Europe, and highlighted that there was no consensus on the definition of retirement and that no measure seemed to be more frequently used than others.

### The Swedish Pension System

The possibilities to retire are embedded in the welfare system and labour market, which vary substantially by country. Sweden, for example, has a universal pension system with a flexible retirement age. Since 2001, there is no statutory retirement age, but income pension can be claimed from age 61 and the guarantee pension from age 65 (König and Sjögren Lindquist 2016). As a consequence, there is a relatively large variation in the timing of retirement which makes it an interesting case to study (Kridahl 2014). The Swedish pension system allows for a stepwise transition with a gradual withdrawal of pension benefits.

In the late 1990s, there were substantial changes to the Swedish pension system (Hagen 2013). Before these reforms, disability, partial pension and early retirement benefits were easily accessible for people with health problems and the most common way to enter retirement before age 65 (Palme and Svensson 2010). This scheme has become more restrictive since the early 2000s, and the benefits are only available if health problems can be attested (Stattin 2005). Disability pension, partial pension and early retirement—which we will collectively refer to as “disability benefits”—are programmes to support those who, because of long-term illness or for other reasons, cannot support themselves through paid work. Disability benefits are closely linked to the old-age pension system as recipients of disability pension are transferred to the guarantee pension when they reach the age of 65. These features of the Swedish pension system render the identification of a specific retirement age complex and challenging.

### Definition of Retirement Age in Studies Using Swedish Data

Multiple studies have been done on retirement in Sweden, using various definitions of retirement and relying on different databases. In studies based on survey data, retirement age is usually self-reported by the survey participants. For instance, in a study using data from the Swedish Panel Survey of Ageing and the Elderly (PSAE), survey participants were asked in a straightforward way to report their actual retirement age (Nordenmark and Stattin 2009). Another study, using the PSAE defined individuals who received 80% or more from the old-age pension or occupational pension benefit in relation to the sum of total income for at least 2 years in a row as retired; individuals who received disability pension were excluded (Örestig et al. 2013). An analysis of the Swedish Level of Living Survey (LNU) used self-reported year of retirement (Kridahl 2014). The HEalth, Ageing and Retirement Transitions in Sweden (HEARTS) study provides four response options to the question “Are you retired (receive old-age pension)?”: (1) no; (2) yes, but still working and consider myself a worker; (3) yes, still working but consider myself a retiree; and (4) yes, full-time retiree. This allows for better estimation of the respondent’s own perception of her retirement transition. Researchers using the HEARTS survey have, based on the item above, applied their own definition of retirement that fit with the objective of the study (Hansson et al. 2018; Henning et al. 2017; Lindwall et al. 2017). In the Swedish Longitudinal Occupational Survey of Health (SLOSH), participants are asked to respond to the most appropriate questionnaire for their current work situation: the one for people working 30% or more of full time during the past 3 months or the one for people who were less than 30% gainfully employed in the past 3 months (Magnusson Hanson et al. 2018). A recent study by Nyberg et al. (2018) using the SLOSH dataset defined retirement age as the date when respondents retrospectively reported having retired with old-age pension.

Also in studies using income register data, retirement age is defined in various ways. It can be defined as the age at which individuals have a reduction in employment earnings and an increase in pension benefits; this type of definition is called *source-of-income* in previous studies. In the literature, there are two different ways of using source-of-income regarding disability benefits. Three studies, using Swedish administrative population register data, include disability benefits in the annual employment earnings along with income from employment and self-employment (Kridahl 2017; Kridahl and Silverstein 2017; Statistics Sweden 2011a). Other studies (Barban et al. 2017; Stenberg et al. 2012; Svensson et al. 2015) include the disability benefits as a pension income. In all of the six studies using register data, individuals were defined as being retired if they received more than 50% of their annual earnings in the form of pension benefits.

A study by Palme and Svensson (2004), also using Swedish administrative population register data, examined two definitions of full-time retirement: first, *source-of-income*, which defines individuals as being retired if, in a particular year, they received more than 80% of their income from public benefits such as state old age pension, occupational pension, disability pension or unemployment insurance. It is very likely that labour income exceeds 20% of total income the year a worker retires due to the annual nature of register data. Consequently, Palme and Svensson set the year of retirement at the year before the worker starts to permanently receive less than 20% of his/her income from labour.

Second, using *earnings-from-labour*, they defined individuals as being retired if, in a particular year, they had income from labour of less than a so-called basic amount (BA). The BA is an indexation unit that price adjusts the Swedish income security system. It is set by the government every year but has followed the consumer price index very closely. The BA in 2010 was SEK 42,400 ($6000, 4400 €). As it is likely that the total annual income in the year of retirement exceeds the BA, retirement age is set to the year before income falls below the BA (Palme and Svensson 2004). Comparing the retirement age generated by these two definitions, the authors conclude that the two measures do not strongly deviate for most individuals. Yet the *source-of-income* definition does not consider that some individuals, mainly women, leave the labour force without immediately claiming pension benefits—perhaps because they were living on income from other members of the household. It also does not identify people who claim full-time old-age pension while continuing to work part-time after age 65. Accordingly, they claim that *earnings-from-labour* is more suitable than *source-of-income* (while defining disability benefits as pension benefits) when assessing full-time retirement. In another study, Johansson et al. (2014) used *earnings-from-labour* to define retirement. They defined gainful employment as an annual income that exceeds the BA. When an individual was not gainfully employed for two consecutive years, s/he was considered to be full-time retired. Retirement age was the age in the last year of employment.

These different definitions of retirement age impede the comparison of results from different studies and thus render policymaking in the context of retirement age challenging. Our study fills this gap by providing a comparison of four definitions of retirement age based on survey and register data used in the literature. This allows us to get a sense of whether they lead to different substantial results and thus different interpretations and implications when examining questions related to retirement age.

## Methods

### Data

This study used survey data from the Swedish Level-of-Living Survey (LNU), a nationally representative longitudinal panel survey, and the Longitudinal Integration Database for Health Insurance and Labour Market Studies (LISA), a Swedish administrative population register covering the whole adult population. Personal identification numbers were used to match individual-level data in the two datasets. Unlike many other countries, where it can be difficult to link register data to survey data, Sweden has the unique possibility of linking register data via personal identification numbers.

LNU was initiated in 1968 and has been repeated 5 times, in 1974, 1981, 1991, 2000 and 2010, making it one of the longest-running surveys in the world (Fritzell and Lundberg 2007; Lennartsson et al. 2014). LNU encompasses a random sample of the Swedish population aged 18 to 75 years. Refreshment samples of younger people and immigrants enable the survey to remain representative of the population of Sweden at the time of each survey wave. The response rate is relatively high, varying between 91% in 1968 and 72% in 2010. The principal survey mode is face-to-face interviews in the respondents’ home. The survey covers a broad range of topics such as living conditions, family situation, health, lifestyle and financial resources and has been used for numerous studies on various subjects. The longitudinal design and the broad range of topics make it possible to study how retirement timing is associated with socio-demographic and individual factors

LISA includes all individuals older than 16 registered in Sweden as of 31st of December each year. The database, updated each year with a new annual register data, integrates existing data from the labour market, educational sector and social sector. The information in LISA provides the basis for longitudinal research about gainful employment and periods of unemployment, training, sickness absence, parental leave and retirement. LISA includes information on annual total employment income (including income from self-employment and unemployment benefits), old-age pensions and disability benefits. Data from LISA are valid and highly reliable because they are based on tax records, and LISA data have been used extensively in research (Statistics Sweden 2011b). For this study, we used the 2010 wave of LNU linked to LISA register data covering the period 1990 to 2011. As the income data are based on tax records, we expected the register data to be highly reliable. We restrict the sample to those individuals who were at least aged 50 at retirement. Additionally, we used either the LNU 1991 or 2000 waves to assess covariates before retirement timing depending on the retirement year. For example, if an individual retired in 1999, we used LNU 1991 to assess covariates before retirement; if an individual retired in 2002, we used LNU 2000.

### Definition of Retirement Age Using LNU Survey Data

Definition of retirement age on the basis of LNU 2010 data required the use of more than one survey question. First, respondents were asked “Last week: Did you receive pension, including sickness or part-time pension?” Those who answered positively were then asked “How many years have you been on pension?” On the basis of this information, we created a variable indicating the year of retirement by subtracting the number of years the person had received benefits from the survey year (for example 2010 − 5 = 2005). We then subtracted the person’s year of birth from their year of retirement to estimate their retirement age (for example 2005 − 1942 = 63). This definition of retirement age is called *self-report* (Table 1) and has been used by Kridahl (2014).

**Table 1:** Operationalization of the four retirement age variables

Variable name	Data source	Operationalization	Previous studies
Self-report	LNU survey data	Self-reported age when first receiving pension	Kridahl (2014)
Source-of-income (DaP)	LISA register data	Income from labour earnings includes the individual’s income from salary and self-employment as well as transfers connected to unemployment and labour market measures. Income from pensions includes occupational pension, old-age pension, early retirement pension and disability benefits	Stenberg et al. (2012), Svensson et al. (2015), Barban et al. (2017)
Source-of-income (DaI)	LISA register data	Annual employment earnings include employment income, income from self-employment *and disability benefits*. Annual pension benefits are occupational pension, old-age pension and early retirement pension	Kridahl (2017), Kridahl and Silverstein (2017), Statistics Sweden (2011)
Earnings-from-labour	LISA register data	In each year, a worker is defined as employed if labour earnings from employment or self- employment exceed one basic amount (BA). A worker is defined as retired in the year after the last observation of employment, if it is followed by at least two years of non-employment. The retirement age is the age in the last year of employment	Johansson et al. (2014)

LNU respondents were not asked to specify the type and level of pension(s) they received, which added a certain amount of ambiguity to the responses. Moreover, retirement age is flexible in Sweden, and people are allowed to continue working when receiving pension benefits. Thus, some survey respondents may have used the date they started receiving pension for the first time and others the date they completely ceased labour market participation.

### Definition of Retirement Age Using LISA Register Data

Based on the LISA register data, we created three variables indicating retirement age by using the same definition previously used in Swedish register studies, see Table 1. We use the same terms as used in the literature.

The first register data variable, called *source-of-income* (*Disability as Pension* (*DaP*)), consisted of total employment income, old-age pensions and disability benefits and replicates the definition of Stenberg et al. (2012), Svensson et al. (2015) and Barban et al. (2017). People are defined as retired when their pension income exceeds 50% of their total annual income from labour earnings. Income from labour earnings consists in individual income from salary and own enterprise as well as transfers connected to unemployment and labour market measures. Income from pensions consists in occupational pension, old-age pension, early retirement pension and disability benefits.

The second register variable, called *source-of-income* (*Disability as Income* (*DaI*)) also consisted of total employment income, old-age pensions, and disability benefits. People are defined as retired when their pension income exceeds 50% of their total annual income from labour earnings and disability benefits. The difference from *source-of-income* (*DaP*) is that the disability benefits are defined as income from labour earnings and not as pension. This definition was used in studies by Kridahl (2017), Kridahl and Silverstein (2017) and Statistics Sweden (2011a).

The third variable, called *earnings-from-labour*, uses drop in annual income from labour over two consecutive years to define retirement age and is based on the measure of Johansson et al. (2014). In each year, a worker is defined as employed if labour earnings from employment or self-employment exceed one basic amount (BA). Retirement age is defined as the year after the last observation of receiving at least the BA from employment, if it is followed by at least 2 years of non-employment. For a worker who is not observed in the data during the second year after the last year of employment, 1 year of non-employment is sufficient to be defined as retired.

The *earnings-from-labour* variable represents a drop in labour income, while *source-of-income* (*DaP*) and *source-of-income* (*DaI*) represent the relative change in labour income and pension income. For *source-of-income* (*DaP*) and *source-of-income* (*DaI*), various cut-offs can be used, more precisely when income from pension stepwise exceeds 10-90% and finally 100% of the annual income. We show the mean retirement age using these different cut-offs, but for the correlation and regression analyses, use the 50% cut-off as has been previously done in studies using these definitions.

### Independent Variables

For the regression analyses, we choose common determinants of retirement age, measured before retirement in either LNU 1991 or LNU 2000 depending on year of retirement (Blekesaune and Solem 2005; Carr et al. 2016; De Preter et al. 2013; König and Sjögren Lindquist 2016; Schirle 2010). As the year of retirement can vary between the four definitions of retirement age, the independent variables had to be created separately for each regression model with the four retirement definitions as dependent variables. More specifically, an individual might have retired in year 2000 based on *source-of-income* (*DaP*), but in year 2002 according to *source-of-income* (*DaI*). In this case, the independent variable used as predictor in the model with *source-of-income* (*DaP*) as dependent variable is the LNU 1991, but in the model with *source-of-income* (*DaI*) as dependent variable is based on LNU 2000. The variables used were sex, age and years of education. Self-rated health was assessed with the question: “How would you assess your general state of health?”, response alternatives were “good”, “neither good nor bad” and “bad”.

Job demands at pre-retirement work consisted of two self-reported items measuring psychological workload and time pressure: “Is your work psychologically taxing/demanding?” and “Is your work hectic?” Participants who answered no to both items were categorized as having low job demands; those who answered yes to one item were categorized as having medium job demands. Those who answered yes to both items were categorized as having high job demands.

Adverse physical working conditions was addressed with items regarding sweating daily at work; the work being physically demanding in any way; having to rush; doing the same job repeatedly and working in uncomfortable body positions; heavy lifts at work; being exposed to loud noise; being exposed to gases; and finally being exposed to poisonous materials, acid or explosives. This variable ranged from 0 (not exposed) to 17 (exposed to all).

### Statistical Analyses

To compare our measure of retirement age in survey data with those created based on the register data, we apply methods typically used for the assessment of measurement error (Bound et al. 2001). In contrast to the methodological literature on how to replace error-prone measures, our analysis does not aim at indicating that survey data is less reliable than register data (Alwin et al. 2014; Uhrig and Watson 2017; van de Pol and de Leeuw 1986). Instead, our analysis strives at showing how different definitions of retirement age compare to each other and lead to different outcomes when doing research on retirement age.

A first step in this procedure is to evaluate the average magnitude of the measurement error (Bound et al. 2001). This is done by calculating the mean and the dispersion of the four variables of interest. The differences are tested with two-sample paired *t* test. A second step is to report correlations between the four variables. Third, standardized regression analyses predicting retirement age were carried out separately for the four ways of identifying retirement age. By analysing whether the results differ in terms of effect size, direction and statistical significance, this strategy aims at indicating whether the use of different definitions of retirement age lead to different conclusions about the determinants of retirement age. It does however not indicate whether some socio-economic groups report retirement age in a significantly different way as has been done in some methodological studies (C. H. Kim and Tamborini 2012). All analyses were performed in Stata 15.

For an overview of the descriptive statistics, see Table 4 (Appendix).

## Results

**Fig. 1:**
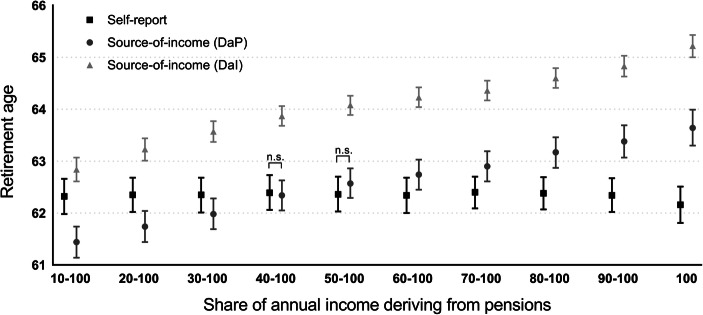
Average retirement age and 95% confidence intervals in the self-report and in different thresholds of source-of-income (DaP) and source-of-income (DaI). Note: The significance of the difference between the averages in the three variables was assessed by means of a two-sample paired *t* test. *T* tests were computed for all pairs at all thresholds (e.g. self-report and 10–100% source-of-income (DaP), self-report and 10-100% source-of-income (DaI), 10–100% source-of-income (DaP) and 10–100% source-of-income (DaI)). Non-significant differences are indicated. The analysis is conditioned on the availability of a measure for all three variables, and on retirement age being 50+ for the source-of-income (DaP) and source-of-income (DaI) variables

We started by comparing average *self-reported* retirement age in the survey data with different thresholds of *source-of-income* (*DaP*) and *source-of-income* (*DaI*) (Fig. 1). *T* tests revealed that the difference between each pair of measures is statistically significant except for the difference between *self-report* and *source-of-income* (*DaP*) at the 40–100% and 50–100% thresholds. This indicates that *source-of-income* (*DaP*) is most similar to *self-report* if people receive about half of their income from pension including disability benefits. A potential interpretation of this result is that individuals perceive themselves as retired and indicate in the survey to be retired when they have reduced their employment to less than half of their standard work week.

The *source-of-income* (*DaI*) variable is different from the other two variables presented in Fig. 1. When only 10% of total annual income stems from pensions, the *source-of-income* (*DaI*) is closest to the *self-report*. The difference between *source-of-income* (*DaI*) and *self-report* increases as a larger share of the annual income derives from pensions. A potential interpretation of this result is that if disability benefits are defined as income from labour, individuals report being retired in the survey as soon as they start receiving any income from pensions (the 10–100% threshold), whereas higher thresholds are reached at higher ages and do not seem to correspond to the individuals’ own perception of being retired. An additional observation is that the retirement age based on *source-of-income* (*DaI*) is about 1.5 years higher than the *source-of-income* (*DaP*) in all thresholds.

**Fig. 2:**
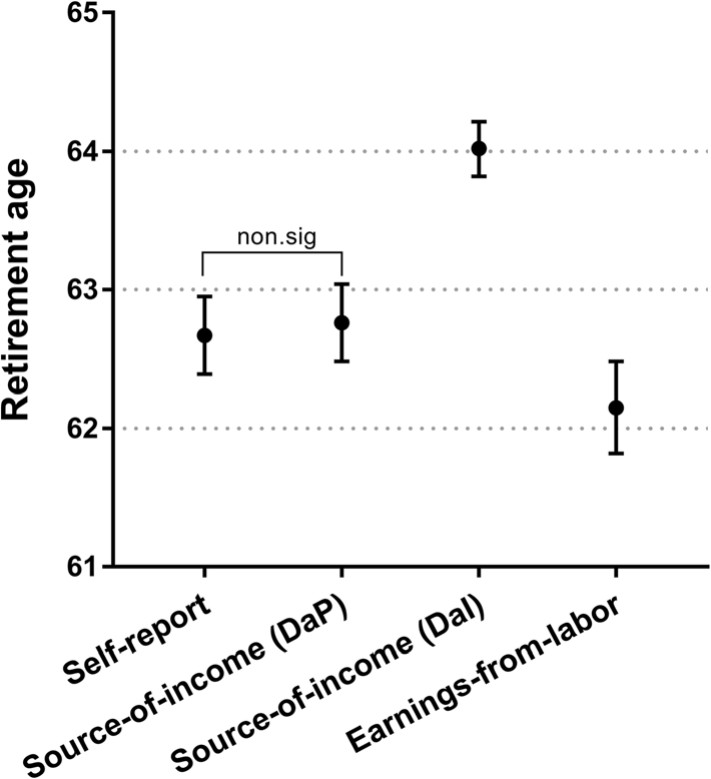
Average retirement age in the self-report, the 50% thresholds of source-of-income (DaP) and source-of-income (DaI), and earnings-from-labour. Note: *n* = 540. *T* tests were computed for all pairs. The significance of the difference between the means in the two samples was assessed by means of a two-sample paired *t* test. The analysis is conditioned on the availability of a measure for all four variables and on retirement age being 50+ for *source-of-income* (*DaP*), *source-of-income* (*DaI*) and *earnings-from-labour*

Figure 2 shows a comparison between the *earnings-from-labour,* the *self-report*, the 50% *source-of-income* (*DaP*) and the 50% *source-of-income* (*DaI*) thresholds. Previous studies have used the 50% threshold for both *source-of-income* (*DaP*) and *source-of-income* (*DaI*) (Barban et al. 2017; Kridahl 2017; Kridahl and Silverstein 2017; Statistics Sweden 2011a; Stenberg et al. 2012; Svensson et al. 2015). *T* tests revealed that each pair of measures is significantly different except for *self-report* versus *source-of-income* (*DaP*). The average retirement age is significantly higher in the *source-of-income* (*DaI*) variable (64 years) than the other variables (62.2–62.8 years).

Table 2 shows the correlations between the same variables as used in Fig. 2. This analysis confirms the results presented in Figs. 1 and 2, in which the *self-report, source-of-income* (*DaP*) and *earnings-from-labour* all strongly correlate. *Self-report* is most strongly correlated with *source-of-income* (*DaP*) (*r* = 0.70), which, in turn, is more strongly correlated with *earnings-from-labour* (*r* = 0.72). The retirement age based on *source-of-income* (*DaI*), where disability benefits are included as labour income, is weakly correlated with the other three definitions of retirement age.

**Table 2:** Correlations between the self-report, source-of-income (DaP) 50–100%, source-of-income (DaI) 50–100% and earnings-from-labour

	Self-report	Source-of-income (DaP)	Source-of-income (DaI)	Earnings-from-labour
Self-report	1			
Source-of-income (DaP)	0.70	1		
Source-of-income (DaI)	0.39	0.49	1	
Earnings-from-labour	0.64	0.72	0.33	1

*n* = 540. The analysis is conditioned on the availability of a measure for all four variables and on retirement age being 50+ for the source-of-income (DaP), source-of-income (DaI) and earnings-from-labour variables

Table 3 shows the results from the regression analyses. The results are consistent in terms of the direction of the associations in the models using *self-report*, *source-of-income* (*DaP*) and *earnings-from-labour*. However, the statistical significance and the size of the associations vary substantially across these three models. We can generally maintain that model 4 using *earnings-from-labour* as a dependent variable provides us with larger effects and more statistically significant results. Overall, models 1, 2 and 4 suggest that women retire earlier than men and the more years of education someone has and the older s/he is, the later s/he retires, although only the associations for education were statistically significant. With respect to self-rated health, these three models indicate that poorer self-rated health is related to earlier retirement. With respect to job demands in the pre-retirement job, we find that individuals with higher levels of demands tend to retire earlier, although the associations were not significant. We find only small and non-significant associations between adverse physical working conditions and retirement age.

**Table 3:** *Z*-standardized OLS regression analysis for four different operationalizations of retirement age

Variables	Model 1		Model 2		Model 3		Model 4	
	Self-report		Source-of-income (DaP)		Source-of-income (DaI)		Earnings-from-labour	
	Coef.	SE	Coef.	SE	Coef.	SE	Coef.	SE
Women (ref. men)	− 0.08	0.04	− 0.16	0.07	0.03	0.08	− 0.12	0.07
Years of education	0.05**	0.02	0.08**	0.03	0.03	0.03	0.10**	0.03
Age	0.11	0.10	0.27	0.17	0.49*	0.19	0.51**	0.18
Self-rated health (ref. good)								
Neither good nor bad	− 0.04	0.05	− 0.13	0.08	0.30**	0.09	− 0.11	0.08
Bad	− 0.14	0.11	− 0.73***	0.19	0.39*	0.18	− 0.49*	0.20
Job demand in pre-retirement job (ref. low)								
Medium	− 0.06	0.05	− 0.07	0.09	− 0.18	0.10	− 0.12	0.09
High	− 0.09	0.07	− 0.08	0.09	− 0.23*	0.10	− 0.16	0.09
Physical working conditions in pre-retirement job	− 0.005	0.02	− 0.02	0.04	− 0.07	0.05	− 0.02	0.04
Constant	0.32	0.15	0.04	0.26	− 0.60	0.29	− 0.17	0.27
*R*^2^	0.04		0.06		0.07		0.06	
*N*	478		478		478		478	

The dependent variables are linear. All variables are *z*-standardized to allow for comparison across models. The analysis is conditioned on the availability of a measure for all variables in the four models and on retirement age being 50+ for the source-of-income (DaP), source-of-income (DaI) and earnings-from-labour variables

*p < 0.05; **p < 0.01; ***p < 0.001, significance levels

Turning to model 3 for *source-of-income* (*DaI*), we observe that individuals with lower levels of self-rated health retire later than individuals with higher levels of self-rated health—results that are rather counter-intuitive. In addition, we find that those with medium and high job demands retire earlier than those with low job demands, the association for high job demands being statistically significant. In line with the *t* test and correlation analysis, the regression analyses indicate that the *source-of-income* (*DaI*) differs from the other three definitions of retirement age.

## Discussion

In this article, we compared the measure for retirement age assessed based on survey data with three different definitions based on register data. The definitions applied are based on previous literature from different disciplines such as sociology, demography, psychology or economics. By replicating four different ways of identifying retirement age used in earlier studies, we assess if and how results from research on retirement may vary, depending on the definition of retirement age. Our analysis is based on a unique dataset linking the Swedish Level of Living Survey with the Swedish Longitudinal Integration Database for Health Insurance and Labour Market Studies, including only those individuals for whom information was available in both data sources.

Our results showed that the measure of self-reported retirement age assessed by means of a survey (i.e. our measure of *self-report*) and a measure of retirement which included disability benefits as a pension income based on register data (i.e. our 50-100% threshold of *source-of-income* (*DaP*)) were very similar. This points to a self-reported retirement age, which is the age at which most of the annual income stems from a source other than paid work. Moreover, it indicates that in Sweden, older people who qualify for disability benefits do not return to paid labour but transfer directly from the social insurance system to the old-age pension system at age 65.

The remaining difference may be due to measurement error induced by social desirability or recall-bias (Bender 2012; Clarke et al. 2008; Drobnic 2002). As the LNU is a face-to-face interview study, respondents may be inclined to provide socially desirable responses, for example to hide early retirement because of health issues (Groves 1990). With respect to recall bias, earlier evidence and the fact that some of the respondents retired many years before their participation in the LNU indicate that recall bias may be present. In fact, in a reliability test of the questionnaire used in LNU 1991, survey respondents were asked to answer the same questions three months later. The analysis showed that the reliability depended on the measure examined (Bygren 1995). Although the overlap was over 90% for measures such as receiving pension benefits the past week or the past year, it was only 47% for the number of years respondents indicated to have received pension benefits (Swedish Institute for Social Research 1998).

Our results also show that if we define retirement age based on the source of income and disability benefits are defined as labour market income (*source-of-income* (*DaI*)), the average retirement age is 1–2 years higher than the self-reported measure or by looking at a drop in earnings from labour. The *source-of-income* (*DaI*) variable thus assesses when people leave the labour market through occupational, income or old-age pensions but hides the fact that many of these people may have exited the labour market earlier through disability benefits. This was also indicated by the result that the 10–100% threshold of the *source-of-income* (*DaI*) variable was most similar to the self-reported measure. In light of the inclusion of disability benefits in the variable *source-of-income* (*DaI*), we found expected results regarding the association between self-rated health and retirement age. Retirement age is *lower* for people with good self-rated health (SRH), which is expected as this variable defines people on disability benefits as being on the labour market

Furthermore, we found that a drop in earnings from employment for at least 2 years (i.e. our measure *earnings-from-labour*) was highly correlated with both the self-reported measure (*self-report*) and the measure that included disability benefits as pension (*source-of-income* (*DaP*)*.* When retirement is defined as withdrawal from the labour market, earnings-related types of operationalizations have been reported as suitable (Denton and Spencer 2009). Being in the labour force adds to the economy’s productivity, hence research concerned with the gross domestic product (GDP) or the ratio between working and non-working population usually use measures based on labour activity.

### Implications

The novelty of our study therefore is that our results indicate how different ways of defining retirement age yield different results in research on retirement age. For instance, researchers who wish to examine whether people in blue-collar occupations retire earlier than people in white-collar occupations may find diverging results depending on the measure of retirement they use. If they use *source-of-income* (*DaP*), they may estimate stronger differences between blue- and white-collar workers than if they use *source-of-income* (*DaI*)*.* This insight should be kept in mind if we compare results across academic disciplines. In fact, depending on the concept that researchers in different fields are using, they may draw different conclusions on processes involved in retirement. While sociologists, psychologists or demographers tend to use subjective measures or measures representing individuals’ *de facto* circumstances (*self-report or source-of-income* (*DaP*)) (see e.g. Kridahl 2014; Nordenmark and Stattin 2009), economists seem to prefer a measure based on income or labour market status (e.g. *earnings-from-labour*) (see e.g. Palme and Svensson 2004; Stenberg et al. 2012; Svensson et al. 2015).

For instance, Palme and Svensson (2004) claim that using *earnings-from-labour* is more appropriate than *source-of-income* (at the 80–100% threshold) when assessing full-time retirement. They argue that people, who partially retired, were living on income from other members of the household or transited from the formal labour market to the informal sector, fulfilled the *earnings-from-labour* criteria of retirement three or more years earlier than the *source-of-income* criteria of retirement. It is worth noting that they used the 80–100% threshold of the *source-of-income*, so an individual’s earnings could be below one BA but still exceed 20–100% of income. This difference might have been smaller if Palme and Svensson would have used the 50–100% threshold for their source-of-income variable. Palme and Svensson highlight that people who retire after the age of 65 according to the earnings-from-labour definition retire two or more years earlier according to the source-of-income. If the objective is to assess full-time retirement, Palme and Svensson conclude that using the earnings-from-labour variable is more suitable than source-of-income because people are allowed to claim pension benefits while still working from the age of 61. One exception might be high-income earners where earnings below one BA might be relatively few hours of work. In such a case, the earnings-from-labour variable may not be a good indicator of full-time retirement (Palme and Svensson 2004). However, if the objective is not to assess full-time retirement but rather the point in time when people start claiming pension benefits, it might be more suitable to use the source-of-income measure that includes disability benefits as pension income (source-of-income (DaP)) at the 50–100% threshold or a self-reported retirement age measure (self-report). We apply this approach in a study on the effect of a prolongation of working life on mortality and health in older adults (Eyjólfsdóttir et al. 2019).

### Strengths and Limitations

A strength of our study is that we use a nationally representative survey that is linked with income register data. Income register data in Sweden covers the entire adult population on an annual basis using tax records and have been shown to be highly reliable and thus provide us with accurate data (Statistics Sweden 2011a). A limitation of our study is that we rely on annual records, but income over the year could vary monthly. The year of retirement could be a year earlier or year later depending on when during the year earnings from labour decreased and the income from pension increased. This limitation also exists in earlier studies using annual records.

### Future Research Directions

An interesting question in the context of our study is how changes in retirement policies affect the measurement of retirement age. The increasing flexibility of retirement systems may for instance further corroborate a trend towards a destandardization of the retirement transition (Barban et al. 2017). This development may dissolve the traditional, and differentiated, phases of the life course consisting in education, employment and retirement, and thus the social identity of retirees, who may still be in work or education, will become increasingly blurred.

The reform of the Swedish pension system between the mid 1990s and the early 2000s—including an abolition of a fixed official retirement age and making disability, partial pension and early retirement benefits accessible only for individuals with severe health problems—provides an excellent setting to study how changes in retirement policies affect the measurement of retirement age. Future research may thus examine whether the measurement of retirement age was affected by the step-wise change of the retirement system.

A possible hypothesis that may be tested is that before the reform, a worker aged 62 who lost her/his job may have identified herself/himself with a retiree due to an access to early retirement benefits, while after the reform, the same person may have identified with a worker due to a loss of this access and possibly due to changing social norms in light of a policy reform that incites an extension of working life. Moreover, since the reform promoted the combination of work and retirement, more older workers may be employed in part-time jobs. Accordingly, the identification with one of the traditional life course phases may become increasingly blurred and the validity of the retirement age measures increasingly impaired. This development will likely call for a new conceptualization of the life course phases and consequently an adjustment of how to measure individuals’ life circumstances in old age (Svensson et al. 2015).

## Conclusions

This paper contributes to the interdisciplinary literature on the conceptualization of retirement age by reproducing and comparing a series of definitions of retirement age. Comparing definitions from register data to self-reported retirement age gives us a better understanding of when in the retirement transition process individuals may start identifying themselves as being retired. The self-reported measure of retirement age resembles most the age in the register data when people receive at least half their income from old-age or disability pension or when they were not gainfully employed for at least 2 years. We therefore provide decision support for researchers working with register data to determine which measure to choose.

## Appendix

**Table 4:** Descriptive statistics

	Self-report	Source-of-income (DaP)	Source-of-income (DaI)	Earnings-from-labour
Sex (%)				
Men	49.3	49.3	49.3	49.3
Women	50.7	50.7	50.7	50.7
Years of education (mean)	11.1	11.1	11.1	11.1
Age in 2010 (mean)	69.4	69.4	69.4	69.4
Self-rated health (%)				
Good	70.9	71.1	69.4	71.5
Neither good nor bad	25.4	25.4	26.0	25.2
Poor	3.8	3.6	4.6	3.4
Job demands in pre-retirement job (%)				
Low	28.7	30.2	36.1	29.1
Medium	26.3	35.6	32.5	36.6
High	35.0	34.2	31.5	34.4
Adverse physical working conditions in pre-retirement job (mean)	2.9	2.9	2.6	3.0

*n* = 478; the sample is the same as in the regression analysis (Table 3). The analysis is conditioned on the availability of a measure for all variables in the four models in Table 3 and on retirement age being 50+ for the source-of-income (DaP), source-of-income (DaI) and earnings-from-labour variables
